# The Role of ZnP_2_ Nanoclusters in the Vibrational Properties of Cd_*x*_Zn_(1 − *x*)_P_2_ Solid Solutions

**DOI:** 10.1186/s11671-016-1635-y

**Published:** 2016-09-22

**Authors:** K. Shportko, T. Shoukavaya, V. Trukhan, J. Baran, S. Starik, E. Venger

**Affiliations:** 1 Lashkarev Institute of Semiconductor Physics, National Academy of Sciences of Ukraine, Nauki av. 45, Kyiv, 03028 Ukraine; 2 Scientific and Practical Center for Materials Science, National Academy of Sciences of Belarus, P. Brovki str. 19, Minsk, 220072 Belarus; 3 Institute of Low Temperature and Structure Research, PAS, 2 Oko’lna Street, P.O. Box 1410, 50-950 Wroclaw 2, Poland; 4 Bakul Institute for Superhard Materials, National Academy of Sciences of Ukraine, Avtozavodska str., 2, Kyiv, 04074 Ukraine

**Keywords:** Nanocluster, Solid solution, Vibrational properties, Optical spectroscopy, 70.78.40.F, 70.63.20.K

## Abstract

This study reports an analysis of the IR reflectance and Raman spectra of Cd_*x*_Zn_(1 − *x*)_P_2_ solid solutions. We have analyzed the effect of the doping of the CdP_2_ single crystal by the ZnP_2_ nanoclusters on the vibrational properties of studied samples: *ε*_0_, *ε*_inf_, phonon frequencies, and strengths. These dependencies might be used as an alternative non-destructive way for the control of the Cd_*x*_Zn_(1 − *x*)_P_2_ composition. The obtained results show that variation of the concentration of ZnP_2_ nanoclusters opens a space to design the tailored material properties for the industrial applications.

## Background

Zinc and cadmium diphosphides ZnP_2_ and CdP_2_ are semiconductors which are characterized by a number of unique properties which make these materials promising for usage in different electronic devices, such as temperature detectors, deflectometers of laser beams, photoconducting cells, magnetic sensors, extenders and stabilizers of laser radiation, and photovoltaic applications [1, 2]. Vibrational properties of ZnP_2_ and CdP_2_ have been previously studied in [3–6] in the wide temperature range. However, vibrational properties of the solid solutions Cd_*x*_Zn_(1 − *x*)_P_2_ have not been studied yet. Doping CdP_2_ by the ZnP_2_ nanoclusters should modify the properties of the solid solutions Cd_*x*_Zn_(1 − *x*)_P_2_. This work is aimed to study the influence of concentration of ZnP_2_ nanoclusters on the vibrational properties of Cd_*x*_Zn_(1 − *x*)_P_2_ solid solutions. In order to achieve this aim, it is desired to understand the role of the ZnP_2_ nanoclusters in the vibrational properties of Cd_*x*_Zn_(1 − *x*)_P_2_ solid solutions to provide valuable information for understanding the electron-phonon interaction and transport properties in these solutions, which influence the electronic device performances.

This paper has the following structure: after the “Background” section which briefly summarizes the previous results and represents the motivation of the work, we describe the experimental procedures, such as preparing the samples, optical spectrum measurements, and their treatment. In the “Results and Discussion” section, we describe the influence of the doping of CdP_2_ by the ZnP_2_ nanoclusters on the vibrational properties of Cd_*x*_Zn_(1 − *x*)_P_2_ by the analysis of the systematic changes in the IR reflectance and Raman spectra. In the “Conclusions” section, we summarize the obtained results.

## Methods

**Fig. 1:**
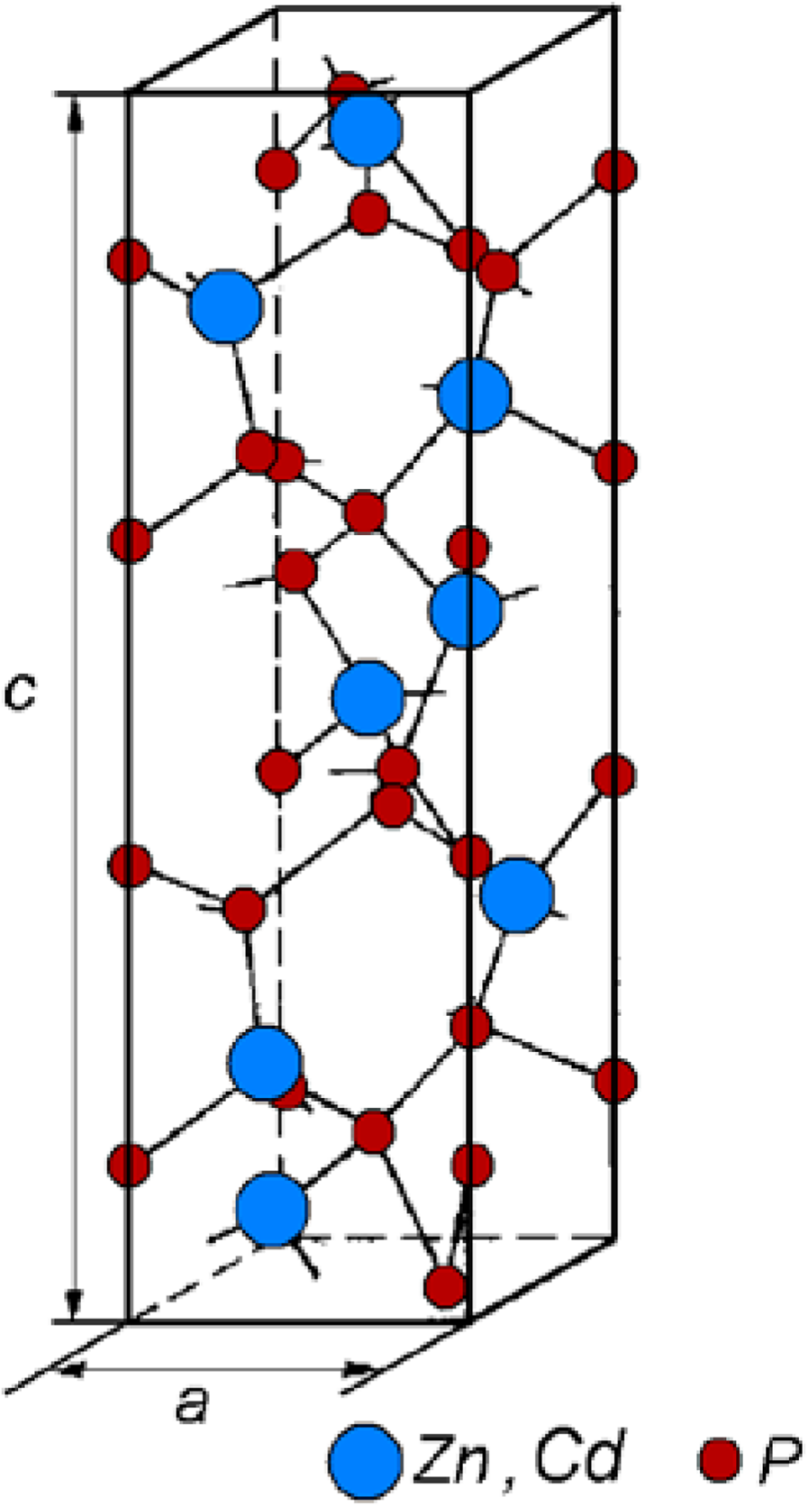
The unit cell of tetragonal CdP_2_ and ZnP_2_ [11]

Tetragonal α-ZnP_2_ and β-CdP_2_ belong to the space symmetry group *P*4_1_2_1_2 (${D}_4^4$) with the following lattice constants: α-ZnP_2_*a* = *b* = 0.50586(7) nm and *c* = 1.8506(4) nm and β-CdP_2_*a* = *b* = 0.52768(7) nm and *c* = 1.9753(4) nm [7]. As one can notice from Fig. 1, where the unit cell of tetragonal CdP_2_ and ZnP_2_ is displayed, each ion of metal M (Zn, Cd) is surrounded by four ions of anion A (P) and each ion A is surrounded by two ions M and two ions A. Ionic radius A is about 0.1 nm, and as A-A distance is 0.21 nm approximately, the chemical bond between anions is rather strong. The ions of anions in the structure of diphosphides form zigzag chains [1]. The ions of metal M are at the center of the deformed tetrahedron and link the chains of anions in a 3D structure. Diphosphides CdP_2_ and ZnP_2_ posses a complex character of chemical bonds: while phosphorus-phosphorus bonds exhibit a covalent character, in metal-anion bond, a proportion of ionic character (from 16 up to 54 % by different estimations) is present [1]. Figure 1 shows also that the elementary cell of CdP_2_ and ZnP_2_ consists of four layers revolved from each other on 90°. Very often, the sequence of packing of layers is broken, and instead of four, it is possible to observe the multiplets of six or five layers.

Polycrystalline CdP_2_ obtained from the initial elements by a two-temperature way were used to grow single crystals of CdP_2_. Ampoules with polycrystalline CdP_2_ were vacuumized till 10^−3^ Pa, soldered, and put into horizontal and vertical resistance furnace. Single crystals of CdP_2_ were grown in the conic side of the ampoule. Constancy of the temperature in the evaporation zone during all the process of growth was achieved by moving the ampoule sideways of the crystallization zone with a speed of 0.6–0.8 mm/h. XRD confirms that grown single crystals of CdP_2_ are single phase.

To obtain Cd_*x*_Zn_(1 − *x*)_P_2_ solid solutions, Zn was deposited on the CdP_2_ single crystal and annealed at the temperature of 650 °C for 600 h. The obtained Cd_*x*_Zn_(1 − *x*)_P_2_ represents a CdP_2_ single crystal with inclusions of crystalline ZnP_2_ with a size of up to 100 nm [1]. Variation of the concentration of ZnP_2_ nanoclusters in the solution was obtained by the variation of the annealing temperature and time. The concentration of ZnP_2_ nanoclusters has been controlled by X-ray fluorescence (XRF). In the present work, we studied two Cd_*x*_Zn_(1 − *x*)_P_2_ samples with *x* = 0.9991 and 0.9997.

In this study, we used a set of samples of single crystals in the shape of plates with a size of 2 mm × 3 mm × 1 mm.

Reflectance spectra of the samples were measured in the range from 100 to 500 cm^−1^ at room temperature, using a Bruker IFS 88 spectrometer with a globar as the radiation source and employing a resolution of 1 cm^−1^ and polarized radiation. We have collected 256 scans in each experiment. Spectra were taken for the ***E*** ⊥ *c* orientation of the electrical vector ***E*** of the IR radiation with respect to the crystal. Raman spectra were measured in the spectral range from 60 to 3600 cm^−1^ at room temperature using a FRA-106 Raman attachment applying the diode pump Nd:YAG laser of ca. 200-mW power and liquid nitrogen-cooled Ge detector for *y*(*zz*)*y* backscattering configuration with the resolution set to 1 cm^−1^ with 256 scans collected in each experiment.

To analyze the reflectance spectra, we used the model of the dielectric function which includes the following summands: *ε*_inf_, which describes the polarizability of bounded electrons and plays the major role in the higher energy range (i.e., core electrons) [8]; phonon contributions were described by Lorentz oscillators with three parameters: the frequency *ν*_*j*_, the damping coefficient *γ*_*j*_, and the oscillator strength *S*_*j*_ [9]. Measured Raman spectra were analyzed in CoRa [10], by modeling the Raman bands with Gauss and Lorentz profiles.

## Results and Discussion

**Fig. 2:**
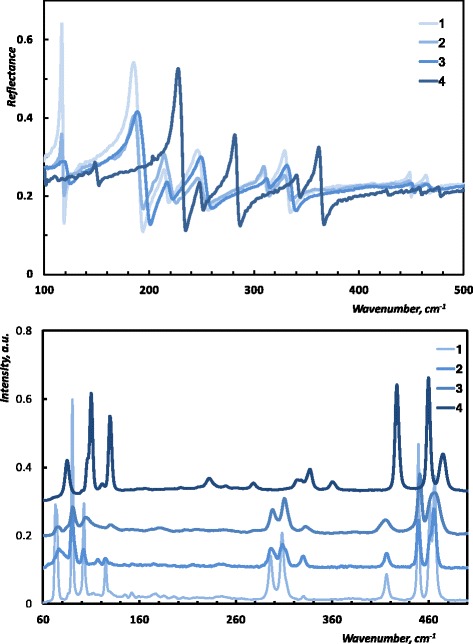
Optical spectra of diphosphides: IR reflectance (***E*** ⊥ *c*), Raman (*y*(*zz*)*y*). *1*—*x* = 1, *2*—*x* = 0.9997, *3*—*x* = 0.9991, *4*—*x* = 0

The vibrational modes of CdP_2_ and ZnP_2_ have the following symmetry types: 9*A*_1_ + 9*A*_2_ + 9*B*_1_ + 9*B*_2_ + 18*E* according to the results of [11]. Modes of the symmetry *A*_2_(*z*) and *E*(*x*,*y*) modes are IR active, whereas *A*_1_, *B*_1_, *B*_2_, and *E* are first-order Raman active. Reflectance and Raman spectra of CdP_2_, two Cd_*x*_Zn_(1 − *x*)_P_2_ (with different *x* values) samples, and ZnP_2_ samples are presented in Fig. 2. In the studied spectral range, we observe eight reflectance and ten Raman peaks. The first visual inspection of the experimental data shows that the obtained reflectance and Raman spectra of the studied samples exhibit a generic pattern. However, one can notice a presence of several differences, such as peaks’ intensities and positions and background values of the reflectance. In the following, we analyze the systematic character of these differences and their correlations with the value of (1 − *x*) which describes the relative amount of zinc in the studied sample.

**Fig. 3:**
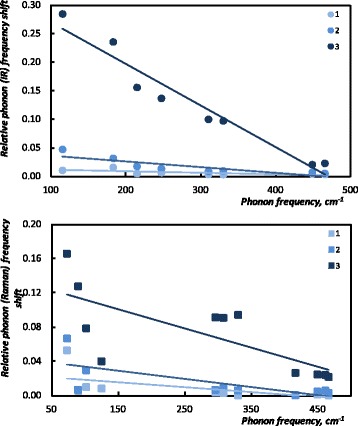
Relative phonon peaks’ (IR and Raman) shift versus the corresponding phonon frequencies of CdP_2_: *1*—*x* = 0.9997, *2*—*x* = 0.9991, *3*—*x* = 0

We start our analysis with the most noticeable feature noticed in the spectra. As mentioned above, the crystal structures of ZnP_2_ and CdP_2_ are similar; therefore, spectra display a generic profile. Qualitative analysis based on the visual inspection of the data convinces that reflectance and Raman peaks shift to the higher wave numbers with increasing the concentration of ZnP_2_ nanoclusters. This can be explained by the difference in masses of Cd and Zn ions: different energies are needed for the excitement of the light Zn and heavy Cd ions. Peaks observed in the reflectance and Raman spectra of the samples of solid solutions originate from the contributions of the corresponding vibrations of Cd and Zn ions. Therefore, to fit these spectra, the frequencies of the corresponding oscillators have been slightly changed with respect to the concentration of ZnP_2_ nanoclusters. In order to perform the quantitative analysis of the phonon frequency changes, we have calculated the relative phonon peaks’ frequency shift $\left({\nu}_j-{\nu}_{j\;{\mathrm{CdP}}_2}\right)/{\nu}_{j\;{\mathrm{CdP}}_2}$ for the studied samples and plotted versus the corresponding CdP_2_ phonon frequencies ${\nu}_{j\;{\mathrm{CdP}}_2}$, as shown in Fig. 3 with linear trend lines for better data visualization. Interestingly enough is that Fig. 3 illustrates also the sensitivity of the low-frequency modes to the change of ZnP_2_ concentration. In diphosphides CdP_2_, ZnP_2_ anion atoms form zigzag chains which penetrate through the crystal [7]. In [12], the low-frequency lattice vibrations have been attributed to the Zn(Cd)-P and Zn(Cd)-Zn(Cd) modes, whereas the high-frequency peaks were assigned to the internal vibrations of the phosphorus chain. Therefore, upon the decrease of the *x*, most changes occur with the low-frequency cation vibrations, whereas the high-frequency vibrations of the phosphorus chain remain mostly unchanged.

**Fig. 4:**
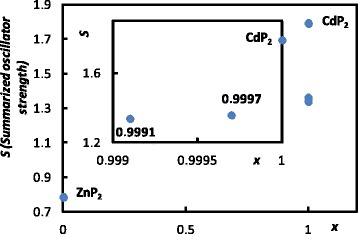
Summarized oscillator strength in the reflectance spectra of Cd_*x*_Zn_(1 − *x*)_P_2_

**Fig. 5:**
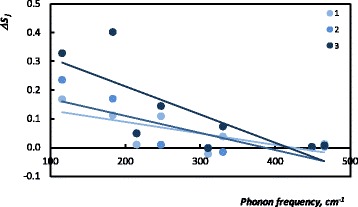
Oscillator strengths versus the corresponding phonon frequencies of CdP_2_: *1*—*x* = 0.9997, *2*—*x* = 0.9991, *3*—*x* = 0

Next, a noticeable feature of the reflectance spectra is the change in the size of reflectance peaks. According to the Lorentz model which was applied to fit these peaks, the parameter *S*_*j*_ called the oscillator strength is responsible for the width of reflectance peaks. Figure 4 represents the systematic change in the summarized oscillator strength on *x*. It is obvious that the summarized oscillator strength steadily increases upon moving towards CdP_2_. As a next step in this analysis, we have looked at the distribution of the oscillator change on the corresponding oscillator frequencies. In Fig. 5, where the Δ*S*_*j*_ is plotted versus the corresponding CdP_2_ phonon frequency, one can see again that low frequency is prone to the main changes of the oscillator strength. We believe that the observed evolution of the oscillators’ strengths could be explained in terms of the electronic polarizability of the vibrating ions. As reported in [13], Cd ions exhibit significantly higher polarizability, and therefore, the replacement of the Cd ions by Zn ones, upon forming the ZnP_2_ nanoclusters, reduces the corresponding dipole moment, which we detected in the reduced oscillator strength. This parameter links the static- and high-frequency dielectric permittivity of the material via a well-known relation:

1 $$ {\varepsilon}_0={\varepsilon}_{\inf }+{\displaystyle \sum_{j=1}^N{S}_j}, $$

**Fig. 6:**
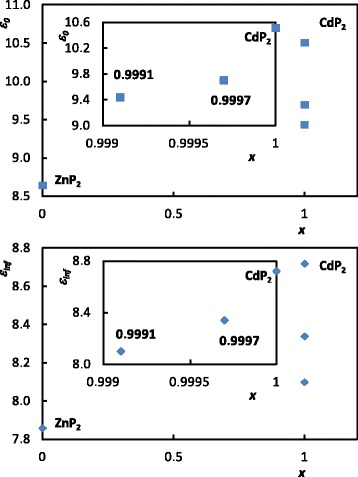
Dependence of the *ε*_0_ and *ε*_inf_ on the concentration of the ZnP_2_ nanoclusters in the studied diphosphides

Interestingly enough is to analyze the impact of the parameter *x* on the dielectric permittivity of Cd_*x*_Zn_(1 − *x*)_P_2_ beyond the phonon range. Whereas in the range of optical phonons the dielectric function is mainly governed by the phonons’ frequency, strength, and damping, above this phonon range and below the optical absorption, the parameter *ε*_inf_ plays a crucial role in the dielectric permittivity of the sample. The *ε*_inf_ is directly linked to the polarizabilites of the material’s components via the Clausius-Mossotti equation [9]. The electronic polarizabilities of the component cations of Cd_*x*_Zn_(1 − *x*)_P_2_ were reported in [13] as 0.46 Å^3^ for Zn and 0.7 Å^3^ for Cd. This explains the higher value of the *ε*_inf_ in CdP_2_ in comparison with the lower value of the *ε*_inf_ in ZnP_2_, as reported in [3, 5, 6, 14]. Thus, as shown in Fig. 6, doping the CdP_2_ by the ZnP_2_ nanoclusters enables one to vary the *ε*_0_ and *ε*_inf_ in the range between the corresponding values of CdP_2_ and ZnP_2_.

## Conclusions

Thus, we performed an analysis of the doping of the CdP_2_ by the ZnP_2_ nanoclusters on the vibrational properties of Cd_*x*_Zn_(1 − *x*)_P_2_ solid solutions. We have shown that ZnP_2_ nanoclusters impact *ε*_0_, *ε*_inf_, phonon frequencies, and strengths. Analysis of these dependences enables one to design the tailored material properties and might be used as an alternative non-destructive way for the control of the Zn concentration in Cd_*x*_Zn_(1 − *x*)_P_2_ solid solutions.
